# Lymph nodes regression grade is a predictive marker for rectal cancer after neoadjuvant therapy and radical surgery

**DOI:** 10.18632/oncotarget.7703

**Published:** 2016-02-25

**Authors:** Jun Li, Jiatian Yuan, Hao Liu, Jie Yin, Sai Liu, Feng Du, Junjie Hu, Ci Li, Xiangke Niu, Bo Lv, Shasha Xing

**Affiliations:** ^1^ General Surgery Department, Affiliated Hospital/Clinical Medical College of Chengdu University, Chengdu, P.R. China; ^2^ General Surgery Department, Second Affiliated Hospital of Jilin University, Changchun, P.R. China; ^3^ General Surgery Department, Xuzhou Central Hospital, Xuzhou, P.R. China; ^4^ Surgical Department of Gastrointestinal Diseases, Beijing Youan Hospital of Capital Medical University, Beijing, P.R. China; ^5^ Internal Medicine-Oncology, Cancer Institute/Hospital, Peking Union Medical College and Chinese Academy of Medical Sciences, Beijing, P.R. China; ^6^ Gastrointestinal Tumor Surgery, Hubei Cancer Hospital, Wuhan, P.R. China; ^7^ Department of Pathology, Affiliated Hospital/Clinical Medical College of Chengdu University, Chengdu, P.R. China; ^8^ Department of Radiology, Affiliated Hospital/Clinical Medical College of Chengdu University, Chengdu, P.R. China; ^9^ Central Lab, Affiliated Hospital/Clinical Medical College of Chengdu University, Chengdu, P.R. China

**Keywords:** lymph node, regression grading, rectal cancer, neoadjuvant therapy

## Abstract

Neoadjuvant therapy (NT) for rectal cancer (RC) reduces primary tumors and involved lymph nodes. While a prognostic value of tumor regression grade (TRG) has been identified, involved lymph node regression grade (LRG) has not been systematically evaluated. Here, we evaluated the association of LRG with oncologic outcomes of RC patients after NT followed by radical surgery. 347 patients with locally advanced RC who received NT and then underwent radical surgery were retrospectively recruited between 2004 and 2011. Response to NT was evaluated by a 3-tier LRG and TRG based on the ratio of residual tumor to fibrosis. LRG was assessed in all patients (LRG 0, 170 patients [49.0%]; LRG 1, 100 patients [28.8%]; and LRG 2, 77 patients [22.2%]). LRG correlated with 5-year distant metastasis and 5-year disease free survival (*p*=0.029 and 0.023, respectively). LRG also correlated with TRG (*p*=0.017). We conclude that the LRG system may be an independent predictive factor of long-term oncologic outcomes of rectal cancer patients after NT and radical surgery.

## INTRODUCTION

Neoadjuvant therapy (NT) in rectal cancer (RC) downstages primary tumors and reduces local recurrence in locally advanced rectal cancer [1, 2]. Large numbers of NT trials have explored the use of tumor regression grading (TRG) as a primary end-point. Various grading systems, including the Manard, Dowrak, Dowark/Rodel, AJCC and MSKCC have been proposed. All of these use the percentage of tumor cells relative to fibrosis. However, TRG scores do not account for the involvement of lymph nodes, which is an important prognostic parameter [3]. In this regard, Perez et al. [4] reported that histologic regression could be observed in mesorectal lymph nodes after NT. Furthermore, studies indicated that TRG of primary tumors may predict lymph node responses [5, 6]. While Caricato et al. [7] demonstrated that LRG correlated with TRG in primary tumors, but they did not examine the impact of LRG on oncologic outcomes. We therefore evaluated the impact of LRG on oncologic outcomes including local recurrence (LR), distant metastasis (DM), and 5-year disease-free survival (DFS), and the LRG correlation with TRG in primary RC tumors.

## RESULTS

### Patient characteristics and association of LRG with clinicopathologic factors

347 patients with locally advanced rectal cancer who received radical surgery in 6-8weeks after NT were identified in this retrospective study. A complete pathologic regression (pCR, ypT0N0) was seen in 46 patients (13.3%). In sum, 4012 lymph nodes were detected in all patients; the mean number of LNs was 11.6±2.3 (range: 1-44 nodes). Of note, 66 (19.0%) patients had the number of LNs less than 10. In total, 676 metastasis mesorectal lymph nodes (16.8%) were found; the mean numbers of positive LNs were 2.1±0.3 and 6.3±1.1 in ypN1-2, respectively. LRG was assessed in all patients (LRG 0, 170 patients [49.0%]; LRG 1, 100 patients [28.8%]; LRG 2, 77 patients [22.2%]). cT stage and cN stage did not predict LRG (*P* =0.815 and 0.432, respectively) (Table 1).

**Table 1:** Association of LRG with pretreatment and tumor characteristics in 347 patients

Variable	LRG 0		LRG 1		LRG 2		Total	*X^2^*	*p*
	No.	%	No.	%	No.	%	No.		
Overall	170	49.0	100	28.8	77	22.2	347		
Age, years									
≤60	88	46.3	58	30.5	44	23.2	190	1.216	0.544
>60	82	52.2	42	26.8	33	21.0	157		
Gender									
Male	105	50.7	62	30.0	40	19.3	207	2.443	0.295
Female	65	46.4	38	27.1	37	26.4	140		
Distance from anal verge, cm									
≤5	93	49.2	55	29.1	41	21.7	189	0.062	0.97
>5	77	48.7	45	28.5	36	22.8	158		
Preoperative CEA									
negative	90	47.4	54	28.4	46	24.2	190	1.261	0.868
positive	70	51.5	40	29.4	26	19.1	136		
unknown	10	47.6	6	28.6	5	23.8	21		
Preoperative treatment									
Preoperative CRT	104	48.8	61	28.6	48	22.5	213	0.039	0.981
Radiotherapy only	66	49.3	39	29.1	29	21.6	134		
cT stage									
cT2	70	50.7	39	28.3	29	21.0	138	2.948	0.815
cT3	58	47.5	34	27.9	30	24.6	122		
cT4	41	48.8	25	29.8	18	21.4	84		
unknown	1	33.3	2	66.7	0	0.0	3		
cN stage									
cN0	90	51.4	45	25.7	40	22.9	175	1.679	0.432
cN+	80	46.5	55	32.0	37	21.5	172		

The association of LRG with histopathologic factors is recorded in Table 2. Radical resection of the primary tumor (R0) was performed in all patients. LRG correlated with TRG score, ypT stage, ypN stage, and venous invasion (*P*<0.05). No significant association was found between LRG and tumor differentiation degree, lymphatic invasion, and tumor deposits after radical surgery.

**Table 2:** Association of LRG with pathological factors after NT and radical surgery

Variable	LRG 0		LRG 1		LRG 2		Total	*X^2^*	*P*
	No.	%	No.	%	No.	%			
Overall	170	49.0	100	28.8	77	22.2	347		
TRG score									
0	46	66.7	23	33.3	0	0.0	69	37.733	<0.0001
1	65	48.9	39	29.3	29	21.8	133		
2	59	38.1	38	24.5	58	37.4	155		
ypT stage									
ypT0	33	47.8	21	30.4	15	21.7	69	31.178	<0.0001
ypT1	60	49.6	40	33.1	21	17.4	121		
ypT2	55	57.9	20	21.1	20	21.1	95		
ypT3	16	50.0	14	43.8	2	16.2	32		
ypT4	6	20.0	5	16.7	19	63.3	30		
ypN stage									
ypN0	155	78.3	0	0.0	43	21.7	198	68.109	<0.0001
ypN1	9	5.9	82	82.0	11	10.8	102		
ypN2	6	12.8	18	18.0	23	48.9	47		
Tumor differentiation degree									
poor	39	48.8	23	28.8	18	22.5	80	0.033	0.997
moderate	59	49.2	35	29.2	26	21.7	120		
well	72	49.0	42	28.6	33	22.5	147		
Lymphatic invasion									
negative	134	48.9	79	28.8	61	22.3	274	0.005	0.997
positive	36	49.3	21	28.8	16	21.9	73		
Venous invasion									
negative	119	49.0	60	24.7	64	26.3	243	11.076	0.004
positive	51	49.0	38.5	28.9	13	12.5	104		
Tumor deposits									
negative	136	48.8	72	25.8	51	18.3	279	5.823	0.054
positive	34	50.0	28	41.2	26	38.2	68		

### LRG as a prognostic factor for DFS

**Figure 1:**
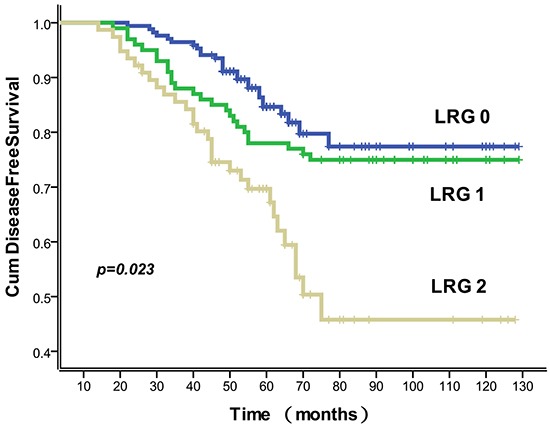
Association of LRG with disease free survival Disease-free survival curves showed a significant relation to LRG. Data for all 347 cases were available. The 5-year disease-free survival rates for LRG 0-2 were 85.9% (146/170), 78.0% (78/100), and 71.4% (55/77), respectively.

The 5-year DFS rate for 347 patients after radical surgery was 80.4%. 34 patients had local recurrence, and 34 patients had distant metastasis. None of the patients suffered from both local recurrence and distant metastasis. 148 (85.9%), 78 (78.0%) and 55 (71.4%) patients with LRG 0, 1, and 2 experienced a 5-year DFS, respectively. TRG correlated with 5-year distant metastasis (*P* =0.035), but failed to correlate with both 5-year local recurrence and 5-year DFS (*P* = 0.531 and 0.576, respectively). LRG correlated with 5-year distant metastasis and 5-year DFS (*P*=0.029 and 0.023, respectively). Disease free survival curve for LRG scores is shown in Figure 1. As listed in Table 3, other factors that correlated with DFS by univariable analysis included the ypT and ypN stage, lymphatic invasion and venous invasion (all *P*<0.05). Using multivariable analysis, the results indicated that two variables including ypT and ypN were independent risk factors for three end-points. LRG was a significant independent predictor of 5-year distant metastasis and 5-year DFS but not for 5-year local recurrence (Table 4).

**Table 3:** Influence of different clinical and pathologic factors on 5-year prognosis after NT and radical surgery

Variables	No. of Patients	Local Recurrence		*P*	Distant Metastasis		*P*	5-Year Disease Free Survival	*p*
		No.	%		No.	%			
Overall	347	34	9.8		34	9.8		80.4%	
Age, years									
≤60	190	19	10.0	0.9	18	9.5	0.077	80.5%	0.983
>60	157	15	9.6		16	10.2		80.3%	
Gender									
Male	207	23	11.1	0.363	22	10.6	0.566	78.3%	0.688
Female	140	11	7.9		12	8.6		83.6%	
ypT stage									
ypT0	69	1	1.4	0.014	1	1.4	0.001	97.1%	0.024
ypT1	121	9	7.4		9	7.4		85.1%	
ypT2	95	11	11.6		8	8.4		80.0%	
ypT3	32	6	18.8		6	18.8		62.5%	
ypT4	30	7	23.3		10	33.3		43.3%	
ypN stage									
ypN0	198	9	4.5	0.001	15	7.6	0.044	87.9%	<0.0001
ypN1	102	13	12.7		9	8.8		78.4%	
ypN2	47	12	25.5		10	21.3		53.2%	
Tumor differentiation degree									
poor	80	13	16.3	0.089	13	16.3	0.083	67.5%	0.468
moderate	120	12	10.0		11	9.2		80.8%	
well	147	9	6.1		9	6.1		87.8%	
Lymphatic invasion									
negative	274	13	4.7	<0.0001	13	4.7	<0.0001	90.5%	0.001
positive	73	21	28.8		21	28.8		42.5%	
Venous invasion									
negative	243	9	3.7	<0.0001	11	4.5	<0.0001	91.8%	0.005
positive	104	25	24.0		23	22.1		53.8%	
Tumor deposits									
negative	279	26	9.3	0.584	25	9.0	0.341	88.9%	0.406
positive	68	8	11.8		9	13.2		75.0%	
Postoperative chemotherapy									
Yes	285	22	7.7	0.014	25	8.8	0.219	83.5%	0.287
No	62	12	19.4		9	14.5		66.1%	
TRG score									
0 (total)	69	4	5.8	0.531	2	2.9	0.035	91.3%	0.576
1 (intermediate)	133	13	9.8		10	7.5		82.7%	
2 (minor and no)	155	17	11.0		22	14.2		73.5%	
LRG score									
0 (total)	170	12	7.1	0.237	12	7.1	0.029	85.9%	0.023
1 (intermediate)	100	12	12.0		10	10.0		78.0%	
2 (minor and no)	77	10	13.0		12	15.6		71.4%	

**Table 4:** Multivariate analysis for three end-points after NT and radical surgery

Variables	5-Year Local Recurrence			5-Year Distant Metastasis			5-Year Disease Free Survival		
	HR	95.0% CI	*P*	HR	95.0% CI	*P*	HR	95.0% CI	*P*
ypT	0.47	(0.29 to 0.80)	0.021	0.55	(0.39 to 0.90)	0.040	0.54	(0.31 to 0.71)	0.033
ypN	2.37	(1.74 to 2.95)	0.006	1.80	(1.45 to 2.02)	0.039	2.58	(2.25 to 3.05)	<0.0001
Lymphatic invasion	1.47	(0.99 to 1.87)	0.083	1.13	(0.90 to 1.28)	0.480	1.03	(0.94 to 1.17)	0.086
Venous invasion	1.12	(0.69 to 1.47)	0.835	0.89	(0.71 to 1.07)	0.713	1.20	(0.99 to 1.47)	0.823
Postoperative chemotherapy	1.24	(0.93 to 1.43)	0.051	–			–		
TRG	–			1.15	(0.90 to 1.29)	0.198	–		
LRG	–			1.37	(1.11 to 1.53)	0.032	1.68	(1.31 to 2.59)	0.027

### Relationship between TRG and primary tumor

All cases were enrolled into the model of correlation test. Spearman correlation test was 3.22 and *p* value was statistically significant (*P* = 0.017). LRG correlated with TRG.

In 198 patients with ypN0, we examined the preoperative CT and MRI images and found that 31 patients had LN+ lesions. Based on this finding, we assumed that NT killed all the tumor cells but pathologists could not find the residual lymph nodes; this group of patients should have good tumor regression in lymph nodes. Thus, these 31 cases (15.6%) were considered as LRG 0. We found that patients with fibrosis (139 cases) and without fibrosis (31 cases) had similar 5-year DFS (83.9% vs. 86.3%, *P*=0.080) (Table 5).

**Table 5:** Association of ypN0 with oncologic outcomes in patients with and without fibrosis after NT and radical surgery

Variables	No. of Patients	No. of Local Recurrence		5-Year Local Recurrence (*p*)	No. of Distant Metastasis		5-Year Distant Metastasis (*p*)	5-Year Disease Free Survival	*p*
		All Cases	5-Year Cases		All Cases	5-Year Cases			
LRG 0	170	13	12		14	12		85.9%	
ypN0 without fibrosis	31	3	3	0.529	3	2	0.814	83.9%	0.080
ypN0 with fibrosis	139	10	9		11	10		86.3%	

## DISCUSSION

Salzer-Kuntschik et al [8] first described tumor regression grade in osteosarcoma after chemotherapy in 1983. In 2002, Bouzorene et al. [9] retrospectively reviewed the resection specimens from 102 patients with locally advanced rectal cancer after preoperative radiotherapy and indicated that tumor regression was a predictive factor for survival. Mandard et al. [10] defined tumor regression in five grades based on residual tumor and fibrosis. In recent years, the TRG grading systems, including the Manard, Dowrak, Dowark/Rodel, AJCC and MSKCC systems, have been proposed. These systems are all based on the percentage of tumor cells relative to fibrosis. However, TRG scores do not account for the involvement of lymph nodes, which are an important parameter of prognosis. In 2007, Caricato et al. [7] reported mesorectal LRG in rectal cancer after NT, but they lacked the long-term data needed to analyze DFS. As far as we know, our study is the first to evaluate the association of LRG with long-term oncologic outcomes.

The results of this study indicate that LRG can predict distant metastasis and DFS, and that LRG is an independent prognostic factor for RC after NT and radical surgery for both DM and DFS. Compared with TRG, we found that LRG correlated with 5-year distant metastasis. Based on the CAO/ARO/AIO-94 trial, Rodel et al. [11] concluded that TRG may be a predictive factor for 5-year distant metastasis. The study group then updated the results and concluded that TRG was a significant prognostic factor for 10-year distant metastasis and DFS [12]. However, our results indicate that TRG may not correlate with 5-year DFS. These differences may be caused by different treatment regimens, including radiation dose, medication used in chemotherapy and pathology practices, as well as differences in duration between NT and surgery. In fact, Kalady et al. [13] reported that patients with an incomplete response at 6 weeks might become pCR at 12 weeks. Accordingly, the interval time between NT and radical surgery was a predominant influence in a pCR, which may impact TRG classification and interfere with the result of the trials. Thus, we consider that TRG alone is not a reliable prognostic factor.

Our study shows that LRG correlates with TRG, as reported previously [5–7]. This indicates that the primary tumor and positive lymph nodes respond similarly to neoadjuvant therapy, which suggests TRG is predictive of the incidence of involved lymph nodes after NT; hence, some authors suggested that TRG might be helpful in selecting patients suitable for a surgically conservative procedure such as local excision [5] or a wait-and-see policy [14–16]. However, in our previous study, we defined a new tumor regression grade (NTRG), which was calculated as the TRG score plus a lymph node score (pN category) and we indicated that NTRG was superior to TRG alone to predict the long-term prognosis of rectal cancer after NT followed by radical surgery [17]. We calculated NTRG using pN stage score not suing LRG, considering that LRG was similar to TRG according to the results of other authors. Interesting, in the present study, we conclude that LRG strongly correlates with TRG, and more surprising, LRG may be useful to assess the long-term prognosis of RC patients. Although our results indicate that LRG may predict long-term oncologic outcomes, some questions remain. First, it is difficult to assess how many of the ypN0 patients with only microscopic LN involvement have really been downstaged. Second, the number of retrieved lymph nodes from patients after NT is lower than in patients treated with radical surgery only. This indicates that NT damages the structure of LNs so that we could not assess whether a small focus of fibrotic tissue found was a pre-treatment normal or metastatic LN. Lastly, pathologists could not distinguish patients with LNs without fibrosis and residual tumor in ypN0 into LRG 0 (complete response) or LRG 2 (no response). Given that we found that patients with and without fibrosis had similar oncologic outcomes, in this study, we enrolled 31 ypN0 patients into LRG 0 (Table 5). But still, we should recognize that complete response in patients with clinical LN+ by MRI but nothing on pathology is not a safe assumption. Besides, does no fibrosis in LN really mean there was any tumor cell before NT, or does LN with fibrosis really mean there is tumor cell ever? These issues should be research in the future study.

Although more studies, including randomized clinical trials are needed, our results indicate that the LRG system may be an independent predictive factor for distant metastasis and DFS of rectal cancer patients after NT and radical surgery.

## MATERIALS AND METHODS

### Patients

The study was approved by local ethic committees of all participating institutions.

We examined records of 347 patients with primary mid-rectal or distal rectal cancer who had received preoperative neoadjuvant therapy followed by radical surgery at four hospitals between June 2004 and October 2011. The study inclusion/exclusion criteria were: (1) rectal adenocarcinoma confirmed by pretreatment biopsy and/or surgical resection with a total mesorectal excision; (2) locally advanced resectable disease (clinical stage II and III) with the distal margin located no farther than 10 cm from the anal verge; (3) no evidence of distant metastasis; and (4) patients underwent neoadjuvant therapy.

### Neoadjuvant therapy schedule

Because there is currently no international consensus with regard to the indications for neoadjuvant chemoradiation therapy, patients managed with preoperative radiochemotherapy or preoperative radiotherapy were identified in our retrospective study. All patients received preoperative radiotherapy (50 Gy/2 Gy/25 f). Among those, 213 (61.4%) patients were concurrently treated with chemotherapy (capecitabine, 825 mg/m^2^/bid), and the rest received radiotherapy alone. All patients received the same capecitabine regimen (1000 mg/m^2^/bid, d1-14, 4-6 cycles) 3 weeks after radical surgery, except 62 (17.9%) patients who rejected chemotherapy due to their age, poor physical condition, or side effects.

### Pathologic examination

All sections of resection specimens were examined specially by local pathologists blinded to patients’ clinical outcomes according to a standardized protocol that included AJCC TNM category, stage grouping, numbers of examined and involved lymph nodes, presence or absence of lymphatic, venous invasion, tumor deposits, TRG and LRG. The lymphatic or venous invasion was identified by morphology using Hematoxylin-Eosin (HE) staining. TDs were assessed using the 3-mm (TNM5) and contour (TNM6) rules.

### Lymph nodes regression grading

Pathologic evaluation of primary tumor regression was performed according to Dworak et al [18], by determining the amount of viable tumor versus fibrotic tissue, which ranged from the lack of tumor regression to complete response with no viable tumor detected. The three groups of TRG and LRG scores were as follows: score 0, total regression (no viable tumor cells; fibrotic mass only); score 1, intermediate regression; score 2, minor regression (dominant tumor mass with obvious fibrosis ≤ 25% of tumor mass), and no regression. Non-metastatic lymph nodes were distinguished from LRG 0 (pCR) by absence of fibrosis. Nodal metastasis regression was evaluated using the same parameters of tumor regression grading referring to each metastatic lymph node. When different LRG scores were identified in one patient, only the most severe score was considered. However, we checked the preoperative CT and MRI pictures and found some patients had LN+ lesions, while pathologists did not found any LN with residual tumor cells and fibrosis (ypN0). Based on that, we assumed that NT killed all the tumor cells but pathologists could not find the residual lymph nodes. This group of patients should have good tumor regression in lymph nodes; thus, these patients (31 cases; 15.6%) were enrolled into LRG 0. Besides, we also found 43 patients (12.4%) with ypN0 but with TRG 2. After evaluating CT and MRI images, the 43 patients were categorized as cN0 and enrolled in LRG 2.

### Follow-up

The follow-up results were collected from all four hospitals databases. The end-point of the follow-up was March 2015. The median time of follow-up was 60 months (26-129 months).

### Statistical analysis

Spearman correlation test was used to assess relationship between TRG and LRG. Local recurrence and distant metastasis analyses were performed for all eligible patients who received R0 resection without distant metastasis found at time of surgery after neoadjuvant therapy. All time-to-event end-points were measured from date of radical surgery. Disease-free survival (DFS) was calculated from radical resection to finding evidence of recurrence and/or distant metastasis. Statistical analysis was performed using SPSS software (version 18). Differences were evaluated with the log-rank test. Analyses for local recurrence and distant metastasis were calculated as cumulative incidences. Mutivariable models were performed using the Cox proportional hazards model. All significant variables in the univariable analysis were included in multivariable Cox regression models in a forward-step procedure. The variables were entered in the order according to clinical relevance into the regression models with increasing complexity, and significance was assessed using analysis of variance analysis. A two-sided *p* value less than 0.05 was considered significant.
